# Experimental study on the effect of grouting reinforcement on the shear strength of a fractured rock mass

**DOI:** 10.1371/journal.pone.0220643

**Published:** 2019-08-12

**Authors:** Cheng Wang, Xuefeng Li, Zuqiang Xiong, Chun Wang, Chengdong Su, Yaohui Zhang

**Affiliations:** 1 School of Energy Science and Engineering, Henan Polytechnic University, Jiaozuo, Henan, China; 2 Henan Collaborative Innovation Center of Coal Work Safety, Jiaozuo, Henan, China; 3 Henan College of Industry & Information Technology, Jiaozuo, Henan, China; Politecnico di Milano, ITALY

## Abstract

To study the strengthening mechanism and effect underlying the reinforcement of a fractured rock mass with grouting, compressive shear tests were conducted with an RMT-150B rock mechanics test system. Prefabricated structural surfaces were strengthened with a new inorganic dual-liquid grouting material at five water–cement ratios (0.6–1.5). The effects of these water–cement ratios of the grouting on the deformation, strength, and failure characteristics of the prefabricated structural surface were analyzed. The results show that reinforcement with grouting significantly influenced the bearing capacity of the structural surface. The shear strength of the structure was significantly improved and the deformation resistance of the structural surface was enhanced. The shear stress–displacement curves, generated in compressive shear tests of the grouting-reinforced structures, were all nonlinear. The shearing process comprised three stages: elasticity, yield, and failure. Decreasing the water–cement ratio of the grouting material weakened the plasticity of the grouted structural surface and enhanced its brittleness. The deformation type changed from plastic slip to brittle shear. The shear strength, cohesion, and angle of internal friction of the grouting-reinforced structural surface increased with decreasing water–cement ratio.

## Introduction

Engineering rock masses are discontinuous, heterogeneous bodies that contain discontinuous structural planes such as cracks and joints. Many studies [1–5] have shown that the mechanical characteristics of the structural surfaces of a fractured rock mass determine its overall mechanical properties. Engineering rock masses typically deform and fail through shear failure, expansion, or by interconnection along a structural plane within the rock body. Eventually, this leads to the failure of the whole structure. Such structural planes are the main factor affecting the continuity and stability of an engineering rock mass [6–8]. Grouting reinforcement technology has been widely used in a variety of engineering contexts to improve the integrity of the engineering rock mass and to strengthen the surrounding rock mass [9–10]. Grouting reinforcement is an effective means to improve the overall strength and stability of an engineering rock mass.

Many scholars have studied the mechanical properties of structural joints within a rock mass [11–12]. Shen et al. [13] analyzed the mechanical properties of a regularly toothed structural plane under shear conditions and established an empirical formula with which to evaluate its shear strength. Other studies reported an improvement of both the shear strength and resistance to deformation of rock fractures after grouting reinforcement. Corresponding increases of the values of mechanical parameters, such as the strength and density of the rock mass, were found [14–15]. Grouting reinforcement exerts a pronounced strengthening effect on fractured rock masses. Normal forces affect the tangential behavior of rock fractures, and the stress strength increases with increasing normal force. Both the strength and stability of a fractured rock mass are increased by grouting reinforcement [16–17]. Since grouting directly changes the mechanical properties of rock fractures it affects the overall mechanical properties of the fractured rock mass [18–19]. Altering the mechanical properties of rock fractures is thus key to influence the physical and mechanical properties of a fractured rock mass [20].

However, until now, research has concentrated on simulating the surface morphology of structural surfaces as well as their shear characteristics after grouting. The mechanical properties of a structural plane strengthened by inorganic grouting materials at different water–cement ratios have not been reported. A new inorganic dual-fluid grouting material has been developed that solidifies and strengthens rapidly. It also offers high permeability, good inject ability, a high calculi rate, and a widely adjustable water–cement ratio. The current study conducted pressure-shear tests with an RMT-150B rock mechanics test system on a prefabricated structural surface that was strengthened with this new inorganic dual-fluid grouting material at five water–cement ratios (0.6, 0.8, 1.0, 1.2, and 1.5). The deformation of this prefabricated structural surface and the effects of grouting at the different tested water–cement ratios on its strength and failure characteristics were analyzed. The results provide information about the mechanism of grouting reinforcement of fractured rock masses and provides guidance on its application in engineering contexts.

## Characteristics of the dual-fluid grouting material

The new inorganic dual-fluid grouting material used for this study comprises a mixture of the two substances A and B. A is superfine sulphoaluminate cement supplemented with a specific proportion of additives, and B is anhydrite and quicklime, also supplemented with additives. When both components are mixed with water at a designed water–cement ratio, the performance of the single component remains stable, without segregation or bleeding over a short period of time (2–6 h). However, the strength increases rapidly, reaching 10 MPa only hours after mixing.

This inorganic dual-fluid grouting material has the characteristics of being fast-setting, providing immediate strength, and having high permeability, a high stone rate, and an adjustable setting time and cementing strength. Preliminary tests indicate that prior to being mixed, A and B slurries do not coagulate, secrete water, or precipitate over the course of 6 h. When both slurries are mixed, this fluidity is lost within 0–5 min, the mixture completely solidifies within 5–15 min, and the pulp stone rate eventually reaches 100% at a water–cement ratio of 0.5–2.0. The strength of the material exceeded 5–21 MPa within 8 h, depending on the water–cement ratio. Table 1 shows the compressive strength of the dual-fluid grouting material.

**Table 1:** Compressive strength test results for the developed inorganic dual-liquid grouting material.

Water–cement ratio	Compressive strength/MPa					
	2 h	4 h	8 h	24 h	3 d	28 d
**0.6**	16.3	20.5	21.7	22.3	22.8	23.2
**0.8**	12.8	13.8	14.6	14.8	15.7	17.5
**1.0**	9.6	10.5	11.4	11.9	12.5	14.7
**1.2**	7.5	8.2	9.6	10.5	11.6	12.7
**1.5**	4.0	4.6	5.2	6.6	7.5	9.3

## Sample preparation and test methods

### Sample preparation

Due to the complexity and changeability of environmental factors, variability and uncertainty exists regarding the structural planes in natural rock masses, which potentially exerts a major influence on the test results. To avoid this influence of the unique features of the sample itself, a cement mortar was used to simulate a rock mass, and a structural surface for grouting reinforcement was prefabricated.

**Fig 1:**
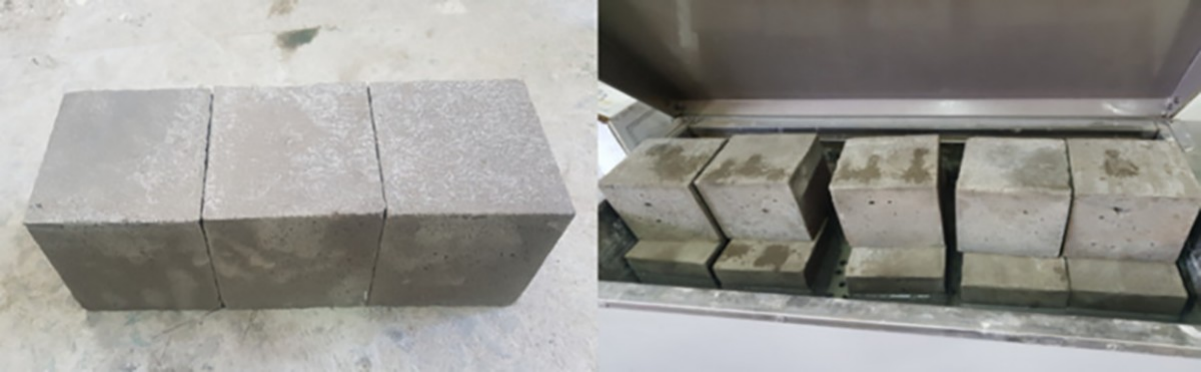
Production and maintenance of cement mortar blocks.

Cement mortar was produced and maintained as shown in Fig 1. Cement (32.5R), river sand, and water were used as mortar materials at sand: cement: water proportions of 2:1:0.55. The 32.5R cement was produced by Jiaozuo Qianye cement Co., Ltd., the river sand was purchased from Wenping building materials, Henan, and the other grouting materials were purchased from Henan Lixing Kechuang Mine Technology Development Co., Ltd. The material was uniformly mixed and poured into 10 cm × 10 cm × 10 cm mortar blocks. After 3 d of curing, a 3 mm diamond was used to cut a prefabricated structural surface through the middle of each block. A dual-liquid grouting system was then used to reinforce the structural surface of the test blocks.

### Grouting process

**Fig 2:**
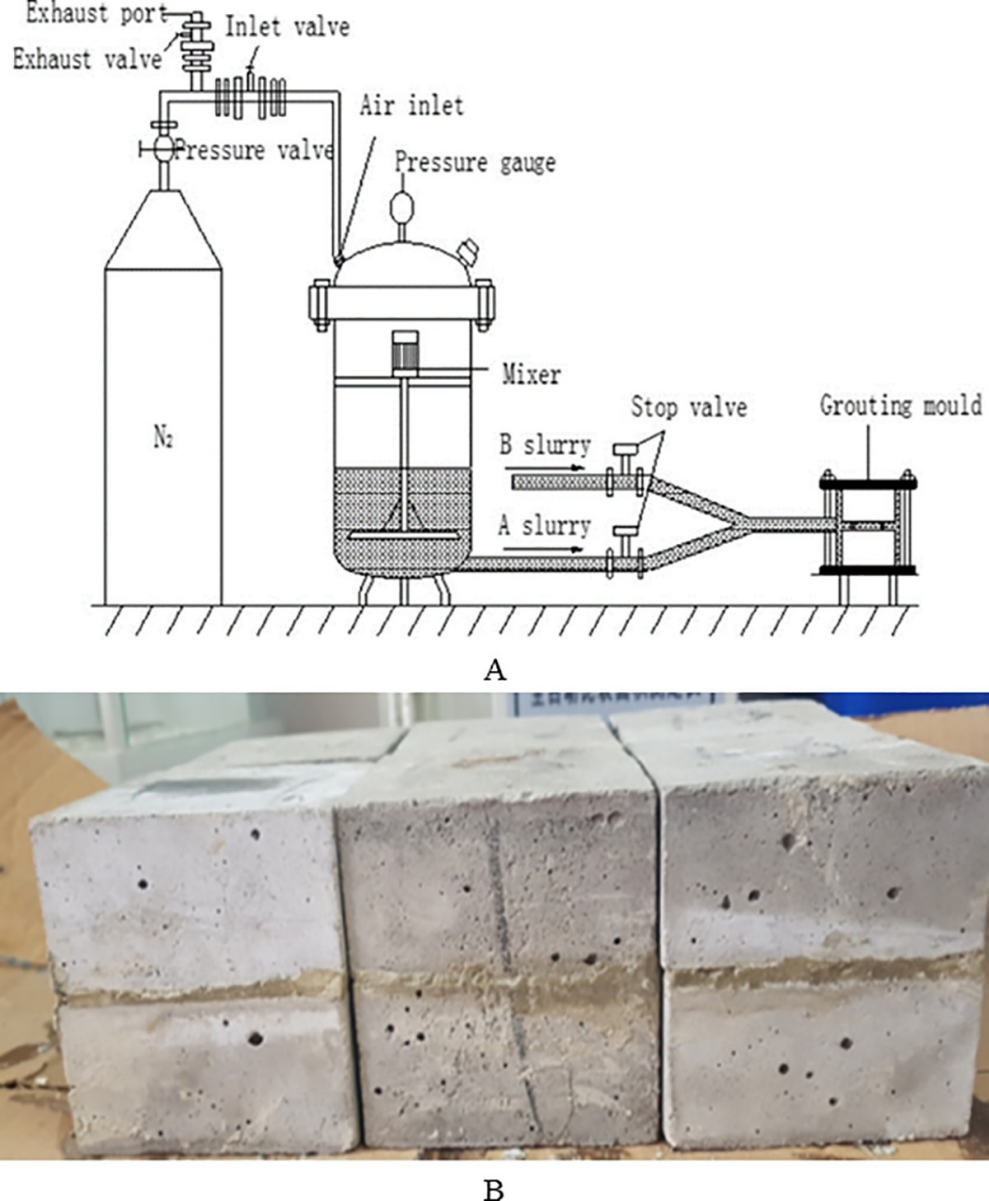
Schematic dual-liquid grouting system and samples after grouting. (A) Dual-liquid fissure grouting system. (B) Samples after grouting.

The grouting reinforcement system for fractured specimens was modified to meet the requirements of fissure-grouting reinforcement with dual-liquid grouting. The system is shown in Fig 2A. Prior to the grouting reinforcement of the prefabricated fracture, the joints of the equipment need to be well-sealed. When the samples are grouted, the vortex mixer continuously stirs the single liquid, the exhaust valve is closed, the intake valve and shut-off valve are opened, and the pressurizing valve is opened, forcing N_2_ into the grouting pressure vessel. Slurry A and Slurry B converge at the T-junction and form a dual liquid for the grouting reinforcement of the structural plane. After the test is completed, the grouted sample is maintained for 28 days.

Five water–cement ratios of 0.6, 0.8, 1.0, 1.2, and 1.5 were selected. Fig 2B shows a number of the grouted samples. Fractured but ungrouted specimens and intact specimens (labeled E1E and E2E, respectively) were also produced and tested for comparison. Seven groups of samples were tested, with five samples per group.

### Test methods

To study the mechanical properties of the rock mass under shear load, orthogonal compressive shear tests were conducted under five normal stress levels. Prior to the compressive shear test, the magnitude of the normal load used in the test was determined. First, the uniaxial compressive strengths were tested. The uniaxial compressive strengths of the three specimens were 23.3 MPa, 26.2 MPa, and 28.5 MPa (with a mean value of 26 MPa). 10%, 15%, 20%, 25%, and 30% of the average value were then taken as normal stresses for the compressive shear tests, resulting in 2.6 MPa, 3.9 MPa, 5.2 MPa, 6.5 MPa, and 7.8 MPa, respectively.

## Results and discussion

### Deformation analysis

**Fig 3:**
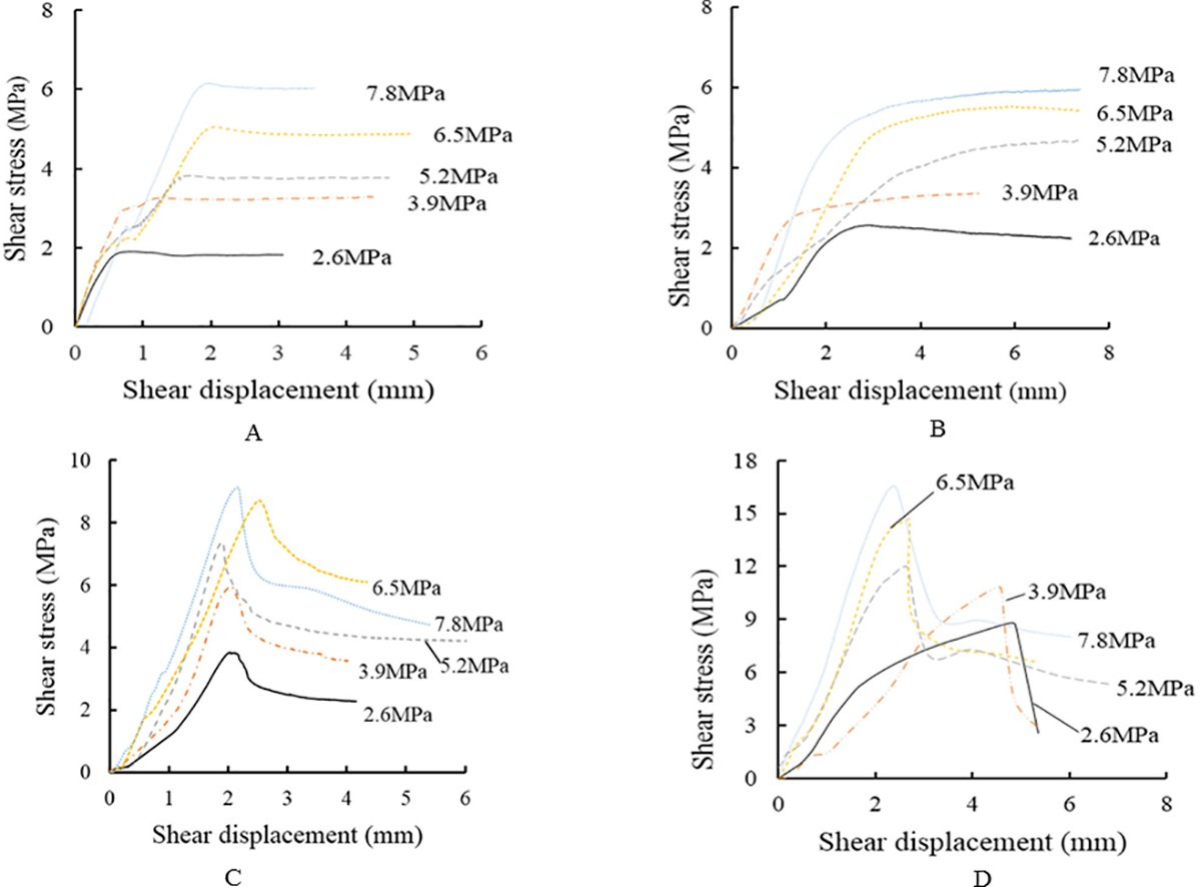
Shear stress–displacement curves with and without grouting. (A) Fractured specimen E1E.(B) Water–cement ratio 1.5. (C) Water–cement ratio 0.8. (D) Complete sample E2E.

Fig 3 shows the shear stress–displacement curves of the structural plane in front and back compressive shear tests of typical grouting-reinforced structures at different levels of normal stress.

Fig 3A shows that sample (E1E), which lacks grouting on its structural plane, has a linear relationship between shear stress and shear deformation during the early stage of loading until the shear stress peaks at a small displacement value. The shear stress then drops slightly from its peak at a clear turning point, and the curve exhibits a yield platform. The peak shear stress tends to stabilize, and the shear displacement continuously increases. Without grouting, the type of shear failure damage at the structural plane is a friction-resisted sliding between structural surfaces in response to normal stress.

The shear failure process of the bonded structural surface is as shown in Fig 3B for a water–cement ratio of 1.5. The curve is smooth throughout the shearing process, without an obvious shear peak. After significant shear displacement, the increasing rate of shear stress decreased and tended toward stability. The shear stress in the peak area remained constant while the shear deformation continued to increase, thus reflecting sliding failure. Due to the high water–cement ratio of the grouting material, its cohesive force is low, and the failure type of the structural surface remains sliding failure.

Fig 3C shows the shear failure process of a bonded structural surface with a water–cement ratio of 0.8. The curve is smooth during the shearing process, showing the three stages of elasticity, yield, and failure. Compared to Fig 3B, the yield process is relatively short, and the curve has a clear shear peak. Prior to the peak value, the grouting material shows characteristics of overall elastic deformation. The grouting material exerts a significant strengthening role during the elastic deformation stage, indicating that the grouting material has a higher degree of adhesion at this ratio. Beyond the peak value, the adhesion of the grouting material decreases and the shear stress drops rapidly. The bearing capacity of the grouting material is still maintained by the frictional force between particles. The overall deformation and failure of the structural plane are characteristic for shear slip.

Fig 3D shows the results of the compressive shear test on a complete rock mass (E2E). The curve displays a clear shear peak, and the overall performance indicates elastic deformation before that peak could be reached. After peaking, the shear stress decreased rapidly. The shear strength of the block itself plays a decisive role during the elastic deformation stage. Then, after the shear failure of the test block, the shear force decreased, and the overall deformation and failure of the structural surface show a characteristic shear slip.

**Fig 4:**
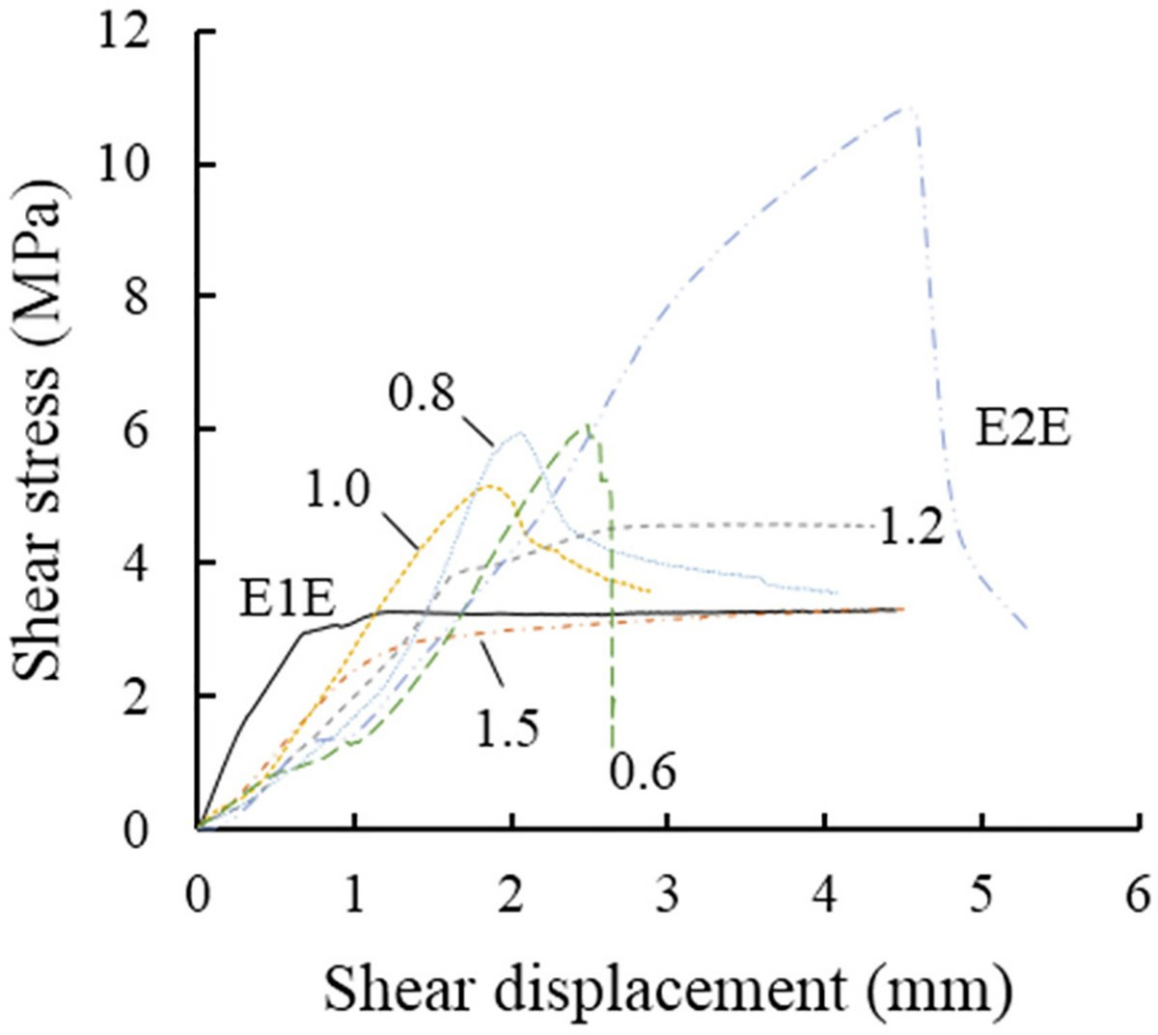
Comparison of shear stress–displacement curves at 3.9 MPa for specimens with and without grouting.

Fig 4 shows a comparison of shear stress–displacement curves before and after grouting under a normal stress of 3.9 MPa. The deformation characteristics of water–cement ratios of 1.5 and 1.2 and E1E are approximately similar, exhibiting no obvious shear peaks. The deformation and failure of the structural plane in these samples show are characteristic for the slip failure type. The curves for water–cement ratios of 1.0, 0.8, and 0.6 and for E2E also have similar characteristics, with a clear shear peak. The curves indicate elastic deformation prior to the peak value and a decrease in shear stress beyond the peak. The stress reduction rate increases with decreasing water–cement ratio. The overall deformation and eventual failure of the structural surface in these samples can be classified as shear slip.

Based on the presented analysis, the mode of deformation of the grouting-reinforced structural plane during a compressive shear test can be concluded to be closely related to the water–cement ratio of the grouting material. With decreasing water–cement ratio, the grouted structural plane is more thoroughly disrupted, thus weakening plasticity, and increasing brittleness.

### Analysis of strength characteristics

**Fig 5:**
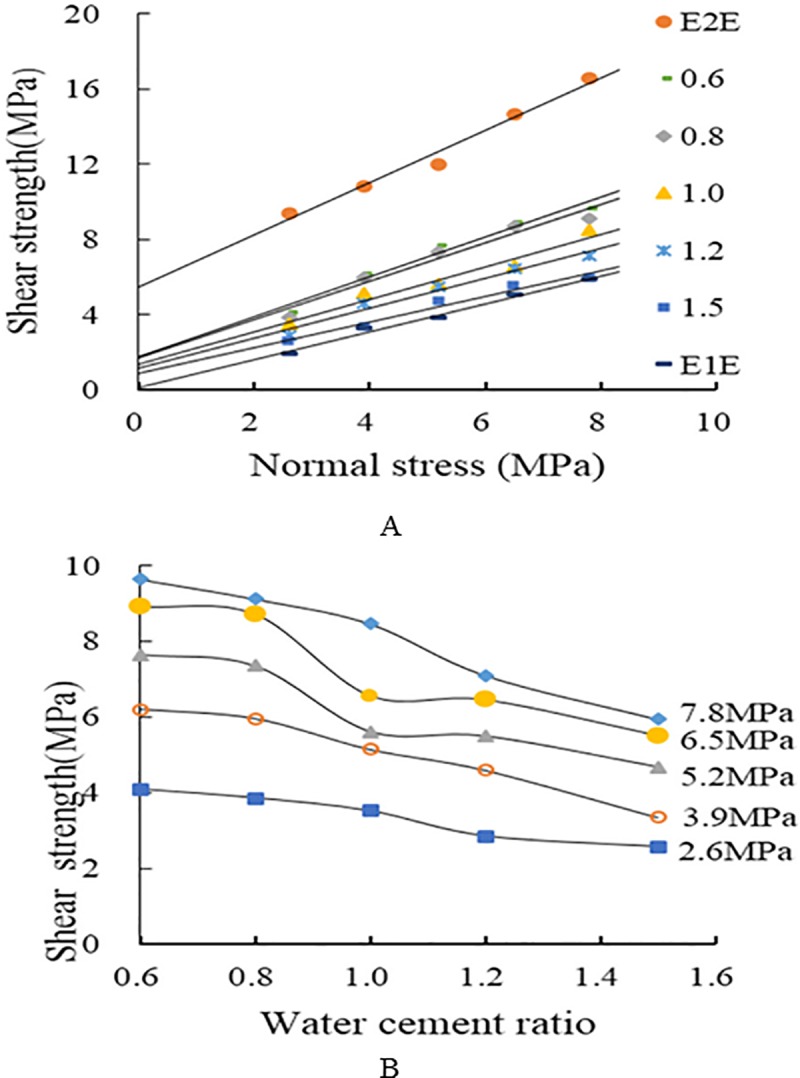
Relationship between shear strength with normal stress and water–cement ratio. (A). (B).

The results of the compressive shear tests are provided in Table 2, and the corresponding shear strength curves are shown in Fig 5. Curves for E1E and E2E are also included for comparison. E2E is a shear test, and E1E as well as the samples with five water–cement ratios are weak-plane shear tests.

**Table 2:** Compression shear test results for structural surfaces.

Normal stress/MPa	Shear strength/MPa						
	E2E	Water–cement ratio					E1E
		0.6	0.8	1.0	1.2	1.5	
**2.6**	9.4	4.1	3.9	3.5	2.9	2.6	1.9
**3.9**	10.8	6.2	6.0	5.1	4.6	3.4	3.3
**5.2**	12.0	7.7	7.4	5.6	5.5	4.7	3.8
**6.5**	14.7	8.9	8.7	6.6	6.5	5.5	5.1
**7.8**	16.6	9.6	9.1	8.5	7.1	5.9	5.9

Fig 5A shows that the shear strength of the grouting-reinforced structural plane is linearly related to the normal stress. In summary, the shear strength of the structural plane increases with increasing normal stress at the same water–cement ratio. Under the same applied normal stress, the shear strength of the complete E2E specimens far exceeds that of non-grouted E1E specimens, and the shear strengths of the grouting-reinforced structural planes lie between both values. This indicates grouting significantly improves the ultimate bearing capacity of a fractured rock mass. For E1E specimens, which lack grouting reinforcement, the upper and lower parts are subjected to horizontal friction due to normal stress compression.

Under the same normal stress condition, the shear strength of the grouted structural plane increases with decreasing water–cement ratio. This becomes more obvious at higher normal stresses, as shown in Fig 5B. This finding can be explained by the fact that when the water–cement ratio of the grouting material is decreased, both the density and cohesion of the paste will increase. Friction will improve due to the higher strength of particles, thus resulting in an increase of the shear strength of the structural plane.

**Fig 6:**
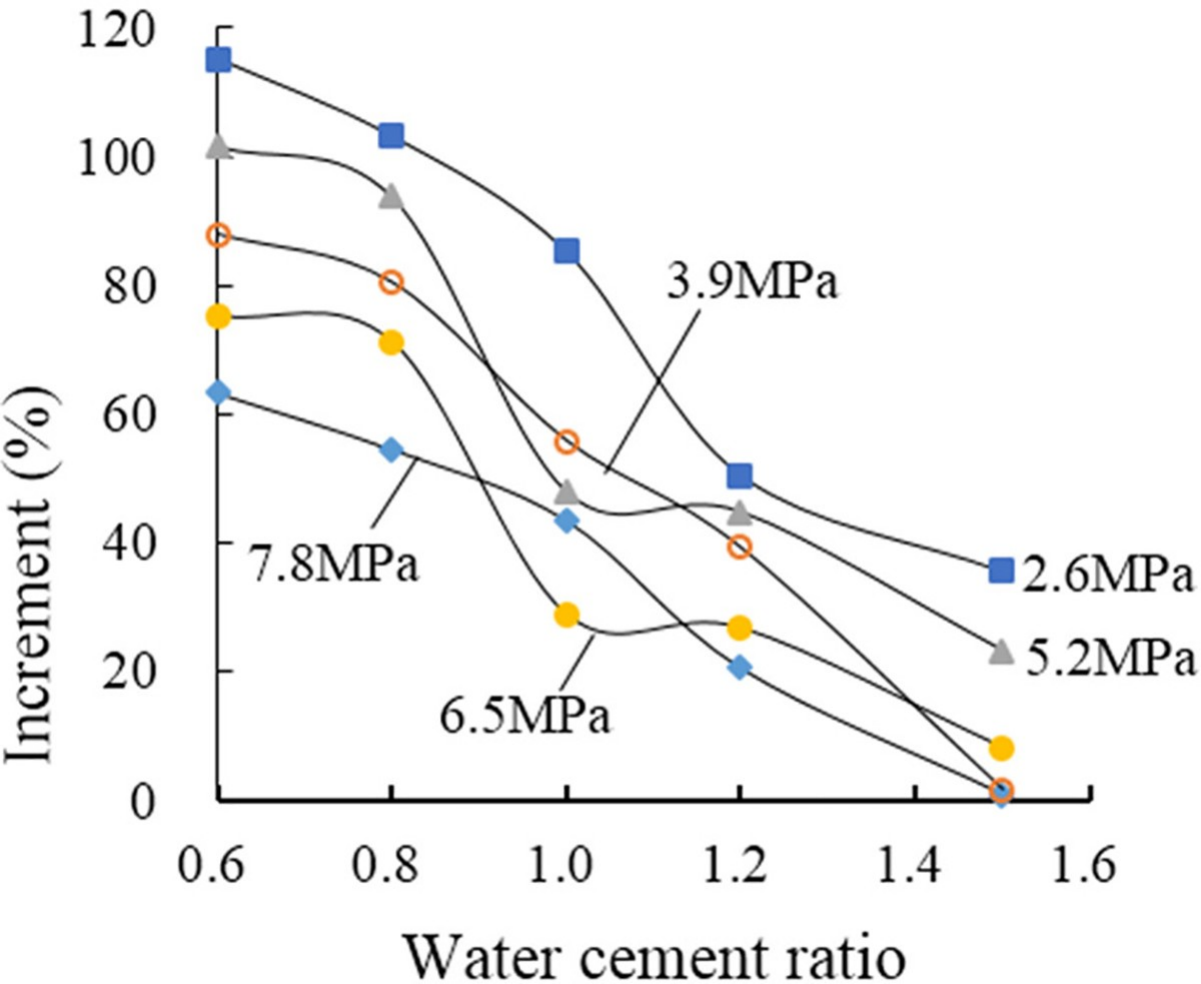
Relationship between increasing shear strength and water–cement ratio.

The reinforcement effect of grouting on the structural plane of a rock mass was assessed via plots of the increment curves of the shear strength of structural planes grouted with various grouting water–cement ratios (Fig 6). The increment of the shear strength ranged between 0.8 and 115.3%. Although the test results were slightly discrete, apart from a drop in the increment under normal stresses of 5.2 MPa and 6.5 MPa and a water–cement ratio of 1.0, the increment of shear strength decreased with increasing water–cement ratio under the same normal stress. These results indicate that the reinforcement effect of grouting is closely related to the water–cement ratio of the grouting material as well as to the normal stress. The overall trend is that the shear strength increment of the grouted structural plane increases when lower normal stress is applied (2.6 MPa) and decreases when a higher normal stress is applied (7.8 MPa). At a water–cement ratio of 1.0, the increment is less at a normal stress of 6.5 MPa than at 7.8 MPa; therefore, its influence on the overall trend can be ignored.

The above analysis indicates that the new inorganic dual-liquid grouting material exerts a marked structural plane reinforcement effect. This reinforcement effect is closely related to the water–cement ratio of the grouting material, where the shear strength with grouting reinforcement improves with decreasing water–cement ratio. When the proportion of cement is relatively high (1.5), the shear strength of grouting-reinforced specimens at higher normal stress (7.8 MPa) is approximately equal to that of non-grouted (E1E) specimens. In response to an increase of normal stress from 2.6 MPa to 7.8 MPa, the shear strength increased by 35.6%, 1.7%, 23.1%, 8.1%, and 0.8%, respectively. At a low water–cement ratio (0.6), the shear strength increased by 115.3%, 87.9%, 101.6%, 75.1%, and 63.4%, respectively, in response to an increase of normal stress from 2.6 MPa to 7.8 MPa. These results indicate that the water–cement ratios for the new inorganic dual-liquid grouting material can be divided into three ranges on the basis of their reinforcing effect: 0.6–0.8, where the effect readily becomes apparent, 1.0–1.2, when the effect is intermediate, and 1.0–1.2, when the effect is poor.

Table 2 and Fig 4A show that the shear strength of a grouting-reinforced structure is linearly related to the applied normal stress, which is in accord with the Coulomb strength criterion.

According to the Coulomb strength criterion:

τ=σtgφ+c (1)

where τ represents the peak strength, φ represents the angle of internal friction, and c represents the cohesion.

Formula (1) was used to regress the data in Table 2, which yielded the strength criterion and correlation coefficient of reinforced structural surfaces with different water–cement ratios. These results are shown in Table 3. Without grouting (E1E), the cohesion at the structural plane was 0.14 MPa and the angle of internal friction was 36.6°. Strengthening by grouting with various water–cement ratios clearly improved the cohesion of the structural surface. This result shows that the ability of the structural surface to bond could be enhanced by grouting reinforcement. The cohesion after grouting with a water–cement ratio of 1.5 was approximately identical to that of the pre-grouting specimen E1E. However, the cohesive force exceeded that before grouting for all other water–cement ratios.

**Table 3:** Shear strength criteria for grouting-reinforced structures.

NO.	Coulomb strength criterion	*R*^*2*^
**E2E**	τ = *tg*54.4°+5.43	0.98
**0.6**	τ = *tg*46.8°+1.78	0.97
**0.8**	τ = *tg*45.6°+1.69	0.95
**1.0**	τ = *tg*41.1°+1.33	0.96
**1.2**	τ = *tg*38.7°+1.16	0.97
**1.5**	τ = *tg*34.2°+0.85	0.97
**E1E**	τ = *tg*36.6°+0.14	0.99

**Fig 7:**
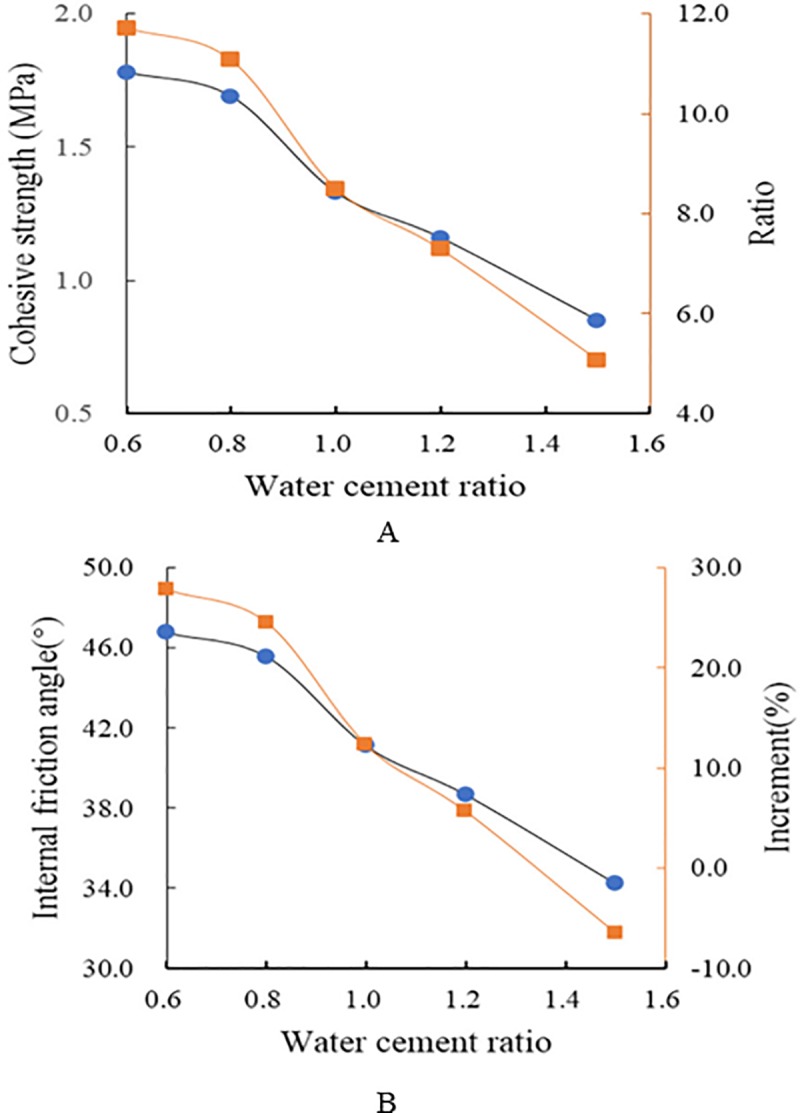
Relationship between the shear parameters of the structural plane and the water–cement ratio. (A). (B).

Fig 7 shows the relationship between the shear parameters of the structural surface and the water–cement ratio. Both values of the shear parameters at different water–cement ratios and their ratio to the values for E1E are plotted. Fig 7 shows that with decreasing water–cement ratio, the values for the shear parameters c and φ of the grouted structural plane increase. Although the matrix material remains the same, the change in the water–cement ratio changes the mechanical properties since the shear parameters of the structural plane are related to the properties of the grouting material. The main reason for this effect is that with decreasing water–cement ratio, both the density and the intermolecular attraction within the grouting material increase, which leads to an increase in cohesion. The friction angle of the structural plane reflects the frictional characteristics of the grouting material. With decreasing water–cement ratio, the volume of internal voids decreases, the intercalation of particles and interlocking and decoupling increase, and in response, the angle of internal friction increases. The ratio of shearing parameters c and φ to those of an ungrouted structure increases with decreasing water–cement ratio. As a result of these changes, the bearing capacity of the reinforced structural surface is increased.

The results of the strength characteristics of grouted structural surfaces indicate that grouting exerts an apparent effect on the bearing capacity of the structural plane. Both the shear strength and deformation-resistance of the structural surface are clearly improved. After grouting, the friction angle at a water–cement ratio of 1.5 remains basically identical to that for the ungrouted condition E1E. With decreasing water–cement ratio, the shear strength, cohesionm and angle of internal friction of the grouting-strengthened structural surface increase. When the water–cement ratio is decreased from 1.5 to 0.6, the cohesion of the grouting-reinforced structural surface is increased by 5.1–11.7 times and the angle of internal friction φ is increased by 5.6–27.8 times.

### Analysis of failure characteristics

**Fig 8:**
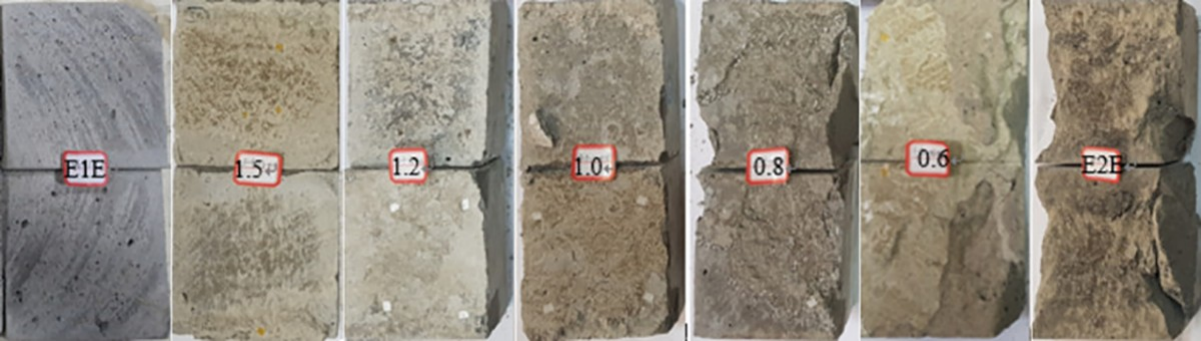
Characteristics of surface compression–shear failure of the grouting-reinforced structures. E1E is a non-grouted specimen and E2E is a complete specimen.

The typical forms of shear failure of the structural surfaces at a normal stress of 6.5 MPa are shown in Fig 8. The upper and lower shear blocks are control blocks. Fig 8 shows that both the upper and lower blocks of sample E1E (without grouting reinforcement) have slipped past each other. After the test, the upper and lower blocks remain intact, but sliding failure has occurred, leaving obvious friction marks on the surface. At a water–cement ratio of 1.5–1.0, signs of the action of grouting friction after shear failure can be found, and the structural plane has mainly failed via slip failure. The likely cause is that the strength of the stone body of the grouting material is low; therefore, the upper and lower shear blocks remain intact after compressive shear failure and shear slip failure occurs within the grouting layer between upper and lower shear blocks. At a water–cement ratio of 0.8, the failure mode of the structural surface changed markedly from that where the water–cement ratio is 1.0–1.5. The failure mode is now shear slip. This is because the self-strength of the stone body of the grouting material is higher, and its bonding ability is stronger. After the compressive shear test, both the upper and lower shear blocks show shear failure adjacent to the bonded surface. This shear failure has mainly occurred at the grouting level; however, the bonded surfaces of the upper and lower shear blocks were also damaged, with shards broken off and expansion on both sides.

At a water–cement ratio of 0.6, the strength of the stone body of the grouting material increased further. The bond between the sample and the grouting material commonly survives the failure of the structure plane. The grouting layer and the upper and lower shear blocks are extensively damaged. Bulge and sag shear blocks comprise about a third of the shear area. The failure of the structural plane is mainly shear-type. The compressive shear failure of the entire E2E specimen is also of shear type, integral failure occurred on the inner surface, and the side of the specimen shows split failure. Tensile failure also occurred locally in the upper and lower blocks.

The following can be concluded from this analysis of the failure characteristics of grouted structural surfaces. The failure characteristics and the water–cement ratio of grouting show a consistent relationship during surface pressure shear testing of grouting-reinforced structures. When the water–cement ratio exceeds 1.0, the deformation and failure of the grouted structural plane generally occur by plastic slip. However, when the water–cement ratio remains below 1.0, deformation and failure gradually change from the plastic slip-type to the brittle shear-type.

## Conclusions

(1) The shear stress–displacement curves of grouting-reinforced structures are nonlinear during compressive shear testing. The shear process has the following three stages: elasticity, yield, and failure. The deformation and failure characteristics of a structural surface under compressive shear testing are closely related to the water–cement ratio of the material used for grouting. With decreased water–cement ratio, the deformation at the grouted structural surface decreased, its brittleness enhanced, and the deformation of the structural surface gradually changed from plastic slip-type to brittle shear-type.

(2) Grouting reinforcement exerts a significant effect on the bearing capacity of a structural surface: the shear strength of the grouting-reinforced structure could be clearly improved, and the resistance of the structural plane to deformation could be enhanced. A decrease in the water–cement ratio of the grouting resulted in increases of the shear strength, cohesion, and angle of internal friction of the structural surface. When the water–cement ratio decreased from 1.5 to 0.6, the cohesion (c) of the grouting-reinforced structural surface increased 5.1–11.7 times, and the angle of internal friction (φ) increased by 5.6–27.8 times.

(3) The water–cement ratios adopted for the new inorganic dual-fluid grouting material can be divided into three ranges: a water–cement ratio of 0.6–0.8 yields an obvious grouting reinforcement effect, a water–cement ratio of 1.5 yields a poor effect, and a water–cement ratio of 1.0–1.2 yields an intermediate effect. These differences can be exploited to meet different engineering requirements.
